# Effectiveness of an Education Toolkit Delivered by Soap Operas Among Communities Living in Extreme Poverty in Improving Vaccination Confidence in the Philippines: Protocol for a Cluster Randomized Controlled Trial

**DOI:** 10.2196/77022

**Published:** 2025-10-30

**Authors:** Shishi Wu, Quanfang Dong, Zhitong Zhang, Sharon Pang, Kevin Thorpe, Melinda Kelly, Victoria Haldane, Lincoln Lau, Xiaolin Wei

**Affiliations:** 1 Dalla Lana School of Public Health University of Toronto Toronto Canada; 2 International Care Ministries Pasig City Philippines

**Keywords:** measles, polio, vaccination, education intervention, randomized controlled trial

## Abstract

**Background:**

Measles and polio pose significant public health challenges globally, particularly in low-resource settings such as the Philippines, where vaccine coverage falls short of the World Health Organization’s (WHO’s) targets, with hard-to-reach populations contributing to the “last mile.” This research addresses the “last mile” challenge in routine immunization efforts by bridging the vaccination gap in marginalized populations.

**Objective:**

We describe the implementation of a cluster randomized controlled trial to evaluate the impact of an education toolkit aimed at improving confidence in measles and polio vaccines among communities living in extreme poverty in the Philippines.

**Methods:**

Developed with local stakeholders, our intervention consists of a 10-minute video and vaccination reminders delivered by health trainers. It is embedded within the Soap Opera Trial, a large cluster randomized controlled trial conducted by the International Care Ministries that evaluates a 15-episode soap opera series combining drama with aspirational messages on hope, self-worth, and education, aimed at improving participants’ knowledge and practices in health, hygiene, nutrition, and livelihood. A total of 180 communities with 5400 participants will be randomly assigned to the intervention and control arms. By leveraging an existing community-based education program on health and livelihood run by our local partner, the proposed intervention will be delivered to participants in the intervention arm of the existing program, while those in the control arm will receive standard participatory adult learning sessions on health education. The primary outcome is the first-dose measles-containing vaccine coverage among participants’ children aged 1 year. Secondary outcomes include the 2-dose measles-containing vaccine coverage among children aged 2 to 6 years, polio vaccination coverage among children aged 1 year, and participants’ knowledge of measles and polio vaccines. The absolute differences in these outcomes between the intervention and control arms will be estimated using generalized estimating equations while adjusting for baseline levels and covariates. In addition, we will conduct a process evaluation.

**Results:**

Between January 31 and February 29, 2024, we recruited 66.9% (3613/5400) of the participants for the trial. Data collection is ongoing at the time of manuscript submission.

**Conclusions:**

Findings from this trial will provide critical insights into effective strategies for enhancing vaccine confidence and uptake in marginalized populations. By leveraging community-based approaches and local partnerships, this study aims to improve public health responses to vaccine-preventable diseases and contribute to global efforts to eradicate measles and polio. Furthermore, the findings will inform scalable interventions that can be adapted to similar contexts, potentially reducing health disparities and advancing global health equity.

**Trial Registration:**

ClinicalTrials.gov NCT06218368; https://clinicaltrials.gov/study/NCT06218368

**International Registered Report Identifier (IRRID):**

DERR1-10.2196/77022

## Introduction

### Background

Measles and polio are highly contagious diseases that can lead to outbreaks with serious public health consequences and strain health systems. Over the past century, measles incidence and mortality have dramatically declined due to advancements in nutrition, health care, socioeconomic conditions, and the introduction of a safe and cost-effective vaccine in the 1960s. From an annual incidence of approximately 4 million cases in 1981, measles cases dropped to under 150,000 by 2016, sparking renewed efforts toward global eradication [1]. However, stagnation in global measles vaccination coverage over the past decade has been a cause of concern. With first-dose measles-containing vaccine (MCV) coverage hovering around 86% and <70% for the second dose MCV, these figures fall short of the World Health Organization’s (WHO) recommended 95% coverage necessary to achieve herd immunity and prevent outbreaks [2]. The Global Polio Eradication Initiative’s extensive vaccination campaigns have been remarkably successful since 1988, reducing polio cases worldwide by 99%. This effort has averted an estimated 20 million cases of paralysis in children. Two primary vaccines are used in this global endeavor: the oral polio vaccine (OPV) and inactivated polio vaccine (IPV), with IPV administered through injection. OPV, the more widely used of the two, plays a crucial role in areas with limited access to sanitation and handwashing facilities [3]. As of 2022, global vaccination coverage for polio stood at 84%, with infants receiving 3 doses of the vaccine. Similarly, in countries that continue to use OPV, 84% of infants had received their first dose of IPV. Targeted for global eradication, vaccines have stopped the spread of wild poliovirus in all countries except Afghanistan and Pakistan. Until poliovirus transmission is interrupted in these countries, all countries remain at risk of the importation of polio, especially vulnerable countries with weak public health and immunization services and travel or trade links to endemic countries [3].

In the Philippines, measles and polio vaccines are provided as part of the routine immunization schedule. For vaccination against measles, monovalent measles vaccine (targeting measles alone at 9 months) and measles-mumps-rubella vaccine (providing immunity against measles, mumps, and rubella at 12 months) are primarily offered as part of the national immunization program [4]. For polio vaccination, the national immunization program includes both OPV and IPV, with OPV administered at 6, 10, and 14 weeks to prevent transmission and IPV administered at 14 weeks to provide systemic immunity [5]. Despite aiming for a 95% vaccination rate, the Philippines experienced a decline in measles vaccine coverage before the 2018-2019 measles outbreak [6,7]. The measles vaccine was introduced in 1983, with a second dose added in 2009 [6,7]. The Philippines’ first-dose MCV coverage peaked at 92% between 2004 and 2008 but dropped to 80% in 2016 and 75% in 2018, with measles outbreaks occurring from 2010 to 2011 and in 2013 [8]. In addition to measles, a polio outbreak was declared in the Philippines in 2019, prompting the Department of Health (DOH) and its partners to initiate a comprehensive outbreak response, including mass polio immunization campaigns starting in October 2019. However, polio vaccination rates reported in 2020 and 2021 were around 70%, which is still substantially below the DOH’s target of 95% [9,10].

Despite the DOH’s continuing efforts to improve vaccination coverage through mass immunization campaigns and annual public health initiatives aimed at increasing awareness, challenges persist in fully reaching the WHO targets, especially in the most remote and underserved communities, often referred to as the “last mile” [11-13]. Specifically, vulnerable populations residing in hard-to-reach communities pose a significant challenge for the delivery of vaccination campaigns and services. These populations are also often those living in poverty or of low socioeconomic status [11]. Although data specifically from the Philippines are sparse, studies from various low- and middle-income countries consistently report lower vaccination rates among households with lower socioeconomic status [14-17]. This inequality in vaccination is often observed in rural areas or urban slums, where public health campaigns and interventions are difficult to implement [14]. Another challenge to vaccination programs is vaccine hesitancy, which largely stems from concerns about vaccine safety, importance, and effectiveness. In the Philippines, vaccine hesitancy has been exacerbated by the controversy surrounding the Dengvaxia scandal in 2016, which involved a dengue vaccine that was prematurely introduced by the Government of the Philippines and the WHO and was associated with several deaths among vaccinated children, fueling skepticism and fear among the public regarding the safety and efficacy of vaccines in general [18]. Moreover, the unprecedented disruption caused by the COVID-19 pandemic has temporarily halted routine immunization schedules, further complicating the delivery of essential vaccination services [19,20]. The pandemic’s impact on health care systems has diverted resources and attention, causing delays in routine vaccinations against diseases such as measles and polio, and reversing gains made in previous years [19,20].

### Objectives

To facilitate successful vaccine delivery to the “last mile” in the Philippines, we will implement an education toolkit that was codeveloped with local stakeholders. The toolkit will be implemented in partnership with an existing community engagement intervention, with the aim of enhancing confidence in measles and polio vaccines. The project will evaluate the education toolkit’s feasibility and effectiveness among households in extreme poverty in the Philippines. Specifically, we will assess (1) the effectiveness of the toolkit in improving measles and polio vaccination rates, knowledge, and attitudes concerning measles and polio and (2) the relevance, applicability, and feasibility of the toolkit in the local context.

## Methods

This protocol is reported according to the SPIRIT (Standard Protocol Items: Recommendations for Interventional Trials) guidelines.

### Study Design

We will use a pragmatic, parallel, cluster randomized controlled trial to assess the effectiveness of an education toolkit in improving public confidence in measles and polio vaccines in the Philippines.

### Setting

International Care Ministries (ICM) is a faith-based nongovernment organization in the Philippines that operates community-based programs focused on poverty alleviation with individuals and households experiencing extreme poverty [21]. Leveraging its organizational strengths in community engagement and outreach, its network of churches and communities, and close partnership with local government units and national government departments (eg, the DOH), ICM delivers its programming through 12 bases across the Philippines. Each base is led by an area head who supports 3 branches staffed by a branch head, health coordinators and trainers (focused on health education, promotion, referrals, and services), livelihood coordinators and trainers (focused on addressing socioeconomic needs), and pastor coordinators (focused on identifying potential partner communities) who provide services in communities [21]. The ICM’s core program is called the Transform Program, which consists of 15 weeks of education sessions on health and livelihood delivered by partner pastors and health and livelihood trainers through participatory adult learning sessions in communities. The intervention delivery timeframe of 15 weeks aligns with the Soap Opera Trial and the delivery structure of ICM’s Transform Program, in which weekly sessions are consistently delivered across communities. Within this framework, our vaccine education toolkit is introduced during weeks 12 to 15, with messages focused on measles and polio vaccination. In addition, this 15-week intervention timeframe allows us to build on participants’ sustained engagement with the transform sessions and ensures that the intervention is delivered in a feasible and standardized manner across clusters. A total of 30 participants from households that meet the criteria for extreme poverty will be selected to participate in the Transform Program in each community [22,23].

Our toolkit will be embedded in an ongoing cluster randomized controlled trial, called the Soap Opera Trial, which is designed and run by the ICM, leveraging its community-based Transform Program at 4 bases (Bohol, Cebu, Dumaguete, and Dipolog). The Soap Opera Trial aims to evaluate the impact of a variation of its standard Transform Program, which uses aspirational videos to deliver information to improve food security, livelihood, and health. The health education sessions include information on general hygiene, nutrition, childhood growth and development, and prevention of sexually transmitted infections and tuberculosis. The soap opera video is entitled “First Light” and was produced by the ICM’s media team. It includes drama and plot twists like a typical television show, but highlights lessons about hope, sense of control, self-efficacy, self-worth, and aspirations for children’s education and future income, as well as on downstream economic outcomes such as income, consumption, and food security, which are key behaviors and outcomes that can help people living in poverty take a step out of poverty. ICM is conducting a cluster randomized controlled trial to assess the impact of these soap opera videos on outcomes such as aspirations about the future, investments in children’s education, social capital, health, hope, and economic outcomes. In this trial, the intervention arm receives the soap opera video for 15 sessions in 15 weeks, while the control arm receives the same information through standard lectures over 15 weeks. The “soap operas” were produced by ICM (which wrote the scripts, hired local actors, videographers, and directed the soap operas) and funded by the Global Innovation Fund. The entire series was filmed on location and in the language of the intended audience. The content of the narratives was developed primarily by ICM staff in consultation with the primary investigators of that study and aimed to embed behavioral nudges within the storyline [24]. Table 1 summarizes both trials and illustrates how our Vaccine Confidence Trial will be embedded in the Soap Opera Trial. Our vaccine confidence toolkit and video are introduced to participants in the intervention arm for 4 weeks (wk 12-15), while the participants in the control arm receive standard education in the form of participatory adult learning, which does not contain vaccine confidence education specified in the intervention arm.

**Table 1:** Summary of Soap Opera Trial and Vaccine Education Toolkit trial.

	Soap Opera Trial	Vaccine Confidence Trial
Intervention aims	To evaluate the impact of a variation of their standard Transform Program, which uses aspirational videos to deliver education about food security, livelihood, and health.	To improve the knowledge of and confidence in measles and polio vaccines in communities living in extreme poverty in the Philippines.
Intervention content	The soap opera, which will be shown in a total of 15 episodes, each lasting 15 min, is entitled “First Light” and was produced by ICM’s^a^ internal media team. It includes drama and plot twists such as a typical television show but highlights lessons about hope, sense of control, self-efficacy, self-worth, and aspirations for children’s education and future income, as well as downstream economic outcomes, such as income, consumption, and food security, which are key behaviors and outcomes that can help people living in poverty take a step out of poverty.	The vaccine education intervention contains two components: (1) an educational toolkit on measles and polio vaccination in the form of a 10-min video and (2) reminder messages regarding vaccination. The video includes information on (1) a comprehensive introduction of measles and polio, (2) vaccine safety and efficacy, (3) measles and polio vaccination guidelines and schedules, and (4) myths and misconceptions. In the toolkit video, we will provide information on (1) the definitions of measles and polio, (2) the importance of measles and polio prevention, (3) the side effects of the vaccines, and (4) locations where vaccinations are provided.
Intervention delivery	A total of 15 soap opera videos will be shown alongside the standard Transform Program over 15 wks.	The intervention will be delivered from wk 12-15 of the Transform Program.
Study participant eligibility	The same participants will be screened using a poverty scorecard, loosely based on PPI^b^ developed for the Philippines (by Innovations for Poverty Action, 2014) and self-reported household income. Participants in households that meet the definition of extreme poverty by income (<22 PHP [US $0.38] per person per day) are prioritized for recruitment into the Transform Program. Participants are eligible if they (1) reside in the participating communities where ICM recruits for the Transform Program and (2) meet the criteria and definition of living in extreme poverty based on ICM’s screening. Those who refuse to participate will not be included in the trial.	The same participants will be screened using a poverty scorecard, loosely based on PPI developed for the Philippines (by Innovations for Poverty Action, 2014) and self-reported household income. Participants in households that meet the definition of extreme poverty by income (<22 PHP [US $0.38] per person per day) are prioritized for recruitment into the Transform Program. Participants are eligible if they (1) reside in the participating communities where ICM recruits for the Transform Program and (2) meet the criteria and definition of living in extreme poverty based on ICM’s screening. Those who refuse to participate will not be included in the trial.
Outcome measures	Outcomes on aspirations about the future, investments in children’s education, social capital, health, hope, and economic outcomes.	Primary outcome measures First-dose MCV^c^ coverage among participants’ children aged 1 year Secondary outcome measures First-dose MCV coverage at the family level Coverage of 2-dose MCV among participants’ children aged 2-6 years Polio vaccination coverage (IPV^d^ or OPV^e^) among participants’ children aged 1 year Polio vaccination coverage at the family level Participants’ mean knowledge score on measles and polio vaccines, which will be assessed using a 10-item questionnaire, 6 mos after the Transform Program
Evaluation methods	Preintervention and postintervention survey	Review of individual immunization records for the children in the participants’ immediate family from the Department of Health, before and after the intervention. Preintervention and postintervention surveys (survey questions will be embedded in the Soap Opera Trial survey questionnaire) Semistructured interviews

^a^ICM: International Care Ministries.

^b^PPI: Progress out of Poverty Index.

^c^MCV: measles-containing vaccine.

^d^IPV: inactivated polio vaccine.

^e^OPV: oral polio vaccine.

### Eligibility and Recruitment

Participants are eligible to be included in our trial if they (1) reside in the participating communities where ICM recruits for the Transform Program, (2) meet the criteria and definition of living in extreme poverty based on ICM’s screening, and (3) give informed consent to participate in the health-education sessions, which include the delivery of our toolkit. Those who refuse to participate will not be included in the trial. Participants will be recruited and screened with an ICM poverty scorecard that uses asset-based scoring loosely based on the Progress out of Poverty Index developed for the Philippines and self-reported household income [25]. The items used for computing the score are elicited through in-person enumeration and home visits. Participants in households that meet the definition of living in extreme poverty by income (<22 PHP [US $0.38] per person per day) are prioritized for recruitment into the Transform Program. There are 30 eligible participants in each community to start the ICM Transform Program. In previous programs, 27% of transform participants went to bed hungry at least once per week. All participants are women with specific parental education levels and have an illiteracy rate of 15%. On average, there are 3.3 children per participant’s immediate family, and 1 child per family falls within the vaccination age for measles and polio, which is between 12 months and 6 years. Children in the household are often malnourished. Economic deprivation is a common unifying factor across households.

### Randomization and Blinding

Recruited communities will be randomized into the intervention and control groups in a 1:1 ratio using computer-generated random numbers developed by a research statistician. A total of 180 communities with all eligible participants (≥30 participants per community) will be randomly assigned to the intervention and control arms in a ratio of 1:1. No blinding will be performed because of the nature of our intervention. As our intervention is integrated into the Soap Opera Trial conducted by the ICM, we will adhere to the trial’s established randomization assignments.

### Process

#### Intervention Arm

All participants of the existing trial (ie, 30 participants per community) in the intervention arm will receive a series of 15 soap opera videos, 1 for each week, to complement the 15-week Transform Program’s education sessions, which are the core intervention of the Soap Opera Trial developed and run by ICM. The 15 episodes were designed to be consumed as a series, with the storylines for each character extending throughout the 15 videos. The characters were developed to reflect situations and contexts comparable to those of the audience, increasing the likelihood that they will relate personally to the story and messages. The videos were shown on televisions brought into the communities (ICM also tested projectors, but the screen setup and lighting proved too logistically challenging). Speakers were also brought into each “watch-session,” and a variety of tests were conducted to ensure that the audience would be able to engage properly with the videos. The soap opera is a popular medium in the Philippines and is widely consumed by the target population. According to NGP, a Filipino-based agency, soap operas are important media sources for Filipinos. Given that the eligible participants live in extreme poverty, the soap operas are a strategic medium for health messaging [24,26]. In addition, all participants in the intervention arm will receive our education toolkit intervention from weeks 12 to 15.

The intervention in our Vaccine Confidence Trial contains two components: (1) education on measles and polio vaccination in the form of a 10-minute video and (2) reminder messages regarding vaccination. The education toolkit will contain information and messages about the following four aspects of measles and polio vaccines: (1) a comprehensive introduction to measles and polio, (2) vaccine safety and efficacy, (3) measles and polio vaccination guidelines and schedules, and (4) myths and misconceptions. In the toolkit, we will provide information on (1) the definitions of measles and polio, (2) the importance of measles and polio prevention, (3) the side effects of the vaccines, and (4) vaccination provision locations. The education toolkit will be presented in local languages using a 10-minute video converted from Microsoft PowerPoint slides. The PowerPoint slide deck and accompanying script were collaboratively developed with health educators from ICM. Professional translators have translated the finalized English version of the slide deck and script into the local languages. Subsequently, ICM produced a video featuring a health worker presenting the toolkit, synchronized with the voiceover and PowerPoint slides.

Following the delivery of the video, participants will also receive reminder messages regarding vaccination from health educators from ICM in the savings groups program running in communities for 4 months. For participants whose children are not yet vaccinated, the health educators will remind them about the importance and benefits of measles and polio vaccination. This process will serve as a reminder for parents about the importance of measles and polio vaccination. In the typical Transform Program, approximately 40% to 50% of participants will self-select to form a savings group, which ICM will support for 2 years. In these groups, participants will receive weekly reminder messages about measles and polio vaccination in their meetings for 4 months.

#### Control Arm

In the control group, participants will attend the standard 15-week Transform Program, with education sessions delivered through standard participatory adult learning sessions by health educators from ICM on the topics of income creation, education, food security, health, and resilience, with key messages consistent with the soap opera videos. No specific educational information will be provided regarding measles and polio vaccination, and no specific reminders regarding measles and polio vaccinations will be provided.

### Outcomes

The primary outcome will be the coverage of the first dose of any MCV among the participants’ children aged 1 year. This will be measured at 6 months after completing the Transform Program. Eligible children must be legal dependents of Transform Program participants and reside in the same household. MCV coverage will be calculated as the proportion of children who have completed at least 1 dose of MCV among the total number of eligible children.

Secondary outcomes will include (1) the first-dose MCV coverage at the family level, which will be determined by the number of participants whose eligible children have completed at least 1 dose of MCV by age 1 year (a family will not be considered covered if any eligible child does not receive the vaccine); (2) coverage of 2-dose MCV among children aged 2 to 6 years; (3) the rate of 2-dose MCV coverage at the family level; (4) polio vaccination coverage, calculated as the proportion of children aged 1 year who will have received at least 3 doses of polio vaccines (IPV or OPV); (5) the polio vaccination coverage at the family level; and (6) participants’ mean knowledge and attitudes score toward measles and polio vaccines, which will be measured by a 10-item questionnaire administered at the beginning and end of the Transform Program. Outcomes 1 to 5 will be assessed 6 months after the completion of the Transform Program. We will conduct subgroup analyses by children’s sex and by province. In addition, we will conduct a process evaluation to assess its relevance, applicability, and feasibility using qualitative research methods.

### Sample Size

The first-dose MCV coverage in the control arm is estimated to be 80% in 2023 based on the WHO report [27]. We estimate that the rate will increase by at least 10% after the intervention. To detect this difference with 90% power using 2-sided testing at the 5% signiﬁcant level, assuming a mean cluster size of 5 children aged 1 year per cluster and an intracluster correlation coefficient of 0.12, we estimate that at least 156 clusters (communities; 78 per arm) will be needed to detect the proposed effect size. To maximize the statistical power, we will recruit all 180 clusters participating in the Soap Opera Trial. We assume that 900 children aged 1 year (450 per arm) will be evaluated for the primary outcome. Because there are 30 participants (ie, parents or grandparents) in each community, we will include them all for a total of 5400 participants in the trial activities.

### Data Collection

To collect primary and secondary outcome data, we will review the individual immunization records of children issued by the DOH for both the baseline and endline periods. To supplement the data retrieved from immunization records, we will also conduct pre- and postintervention surveys using structured, closed-ended questions to collect data on study participants’ sociodemographic information, self-reported vaccination status of their children, and their knowledge and attitudes toward measles and polio vaccines.

To assess the self-reported knowledge and attitudes toward measles and polio vaccination, we will distribute a survey to all participants before and after the intervention. In this survey, 2 items with 5-point Likert-scale responses will be used to assess vaccination attitudes, yielding a total score ranging from 1 to 10. The two items are (1) How much do you agree with the statement “Measles and polio vaccines are safe and effective”? and (2) How much do you agree with the statement “My concerns about vaccine-related side effects deter me from getting my children vaccinated”? All the Transform Program participants in both arms will participate in the pre- and postprogram survey. The ICM team will conduct primary data collection as part of their trial operations, and all personally identifiable information will be removed before sharing it with the study investigators. The survey questions will be administered by ICM staff through in-person interviews. In addition, to enhance the accuracy of the data collected, survey administrators from ICM will verify the vaccination status of the participants’ children by inspecting their vaccination cards. All participants of the Transform Program will be asked to complete the survey before and after the Transform Program as part of the ICM’s standard evaluation process. Preintervention surveys will be completed in February 2024, and postintervention surveys will be completed in August 2024. Vaccination data collection will be validated with local government vaccination records by June 30, 2025.

The ICM surveys will be conducted using SurveyCTO, which stores uploaded data on Amazon Web Services servers. All data will then be backed up using Boxcryptor, which encrypts cloud-based files. Personal identifiable data will be shared only after encryption and will be password-protected. Survey participants will provide consent through the ICM’s survey and data collection processes. The ICM’s survey team will conduct primary data collection as part of their program operations, and all personally identifiable information will be removed before being shared with the investigators of this study. The data will only be analyzed at the group and subgroup levels, and individuals will never be isolated during the process.

### Statistical Analysis

The data will be analyzed following an intent-to-treat approach and at the individual level. Sociodemographic characteristics of community members will be summarized descriptively using means (or medians) or proportions. For the primary and secondary proportion-based outcomes, the absolute differences in vaccination coverage between the intervention and control arms 6 months after the end of the Transform Program will be estimated using the generalized estimating equation models with a logit link while adjusting for baseline vaccination levels and covariates, including sex, age, and province. The odds ratio and 95% CI will be calculated to express the treatment effect. Preintervention and postintervention survey scores in knowledge and attitudes toward measles and polio vaccines will be calculated for the 2 arms, respectively. Generalized estimating equation models will be used to assess the effect of the toolkit on the postintervention knowledge and attitude scores, while adjusting for baseline level and covariates. Subgroup analyses by the 4 bases will be performed. Analyses will be conducted using either Stata (StataCorp) or R software (R Foundation for Statistical Computing). A 2-sided *P*<.05 will be considered statistically significant for the primary outcome analysis. For other analyses, *P* values will be computed and interpreted as the level of statistical evidence against prespecified null hypotheses.

### Process Evaluation

The relevance, applicability, and feasibility of our education toolkit will be assessed using qualitative research methods. In the intervention arm, we will purposively identify and recruit relevant toolkit end users, including the health educators and participants of the Transform Program. We will purposively select 30 stakeholders to participate in semistructured interviews within 2 weeks after the end of the Transform Program as in Multimedia Appendix 1. The interview will aim to understand the accuracy and appropriateness of the toolkit content, user perceptions of the relevance of the toolkit, and overall satisfaction with the toolkit.

Interviews will be conducted in person, online, or by phone, depending on the availability and preference of interviewees, by members of the Canadian research team or research staff from ICM. Interviews will be conducted in the preferred language of the interviewee. In cases where interviews are not conducted in English, an interpreter will be hired to assist with interview facilitation. Each interview will begin with an explanation of the study, followed by the delivery of a verbal consent script and signing of the consent document. Consent will be obtained for both study participation and audio recording (the consent process is described in detail in the subsequent section). Each interview is expected to last 40 to 60 minutes with the aid of an interview guide. Interviews will be audio recorded. For participants who consent to study participation but not to audio recording, we will seek consent to take notes during the conversation. Data collected from the interviews will be transcribed from the recordings. We will use a thematic analysis approach following the 6-phase framework of Braun and Clarke: (1) data familiarization through repeated reading of transcripts and noting initial ideas, (2) systematic coding of meaningful features across the dataset, (3) collating codes into initial themes and subthemes, (4) reviewing and refining themes against coded data and the entire dataset, (5) defining and naming themes to capture their essence, and (6) producing the final analytic report [28]. Transcripts will be coded inductively by 2 researchers, with discrepancies discussed and resolved through consensus. The analysis will be performed using NVivo (Lumivero). Throughout the analysis, we will maintain reflexive memos to document assumptions, positionality, and decision-making throughout the analytic process, ensuring transparency and minimizing bias [29]. To strengthen transferability, we will provide descriptions of the study setting, participant characteristics, and contextual factors, enabling readers to assess the applicability of the findings to other contexts [29].

### Ethical Considerations

Ethics approval for this trial was obtained from the Office of Research Ethics at the University of Toronto, Canada (45722) and the ethics committee of ICM (2024-001). Informed consent will be obtained from participants before trial enrollment. The data will be deidentified before analysis. No compensation will be provided to participants.

### Trial Management

The trial will be co-led by XW (University of Toronto) and LL (ICM, Philippines), who will have full access to the trial dataset. To protect the safety and privacy of participants and to ensure that data are collected ethically, stored correctly, and used solely for research, we will establish a data management committee that will be led by external and independent members. In addition, we will form a trial steering committee to oversee the trial design and implementation. Teleconference meetings will be held for both the data management committee and the trial steering committee at the start and midpoint of the study, with the option of additional meetings as necessary. Protocol modifications will be discussed during these meetings.

## Results

This study was funded in 2021 and registered at ClinicalTrials.gov (NCT06218368) on January 23, 2024. Participant recruitment was launched on January 31, 2024, and completed on February 29, 2024. A total of 3613 participants have been recruited for the trial. Data collection is ongoing and is expected to be completed by June 30, 2025, and we plan to disseminate the results by September 30, 2025. We will disseminate the trial results through research articles and policy briefs. We aim to publish our results in leading international medical journals and present them at national and international conferences. The total lifespan of the study will be 23 months, as shown in Table 2.

**Table 2:** Study timeline.

			Study period (months)										
			1-3		4		5-8		9-10		11-20		21-23
**Preparation**													
	Study design	✓											
	Development of research tools	✓											
	Ethics evaluation	✓											
**Interventions**													
	Enrollment			✓									
	Intervention arm			✓		✓							
	Control arm			✓		✓							
**Evaluation**													
	Primary outcome data collection			✓		✓		✓		✓			
	Process evaluation data collection			✓		✓		✓		✓			
	Data analysis and results dissemination											✓	

## Discussion

### Anticipated Findings

Our trial aims to assess the effectiveness and feasibility of an education toolkit developed in collaboration with local stakeholders to enhance confidence in measles and polio vaccines among communities living in extreme poverty in the Philippines. Conducted in partnership with ICM, the trial will use a cluster randomized controlled design across 4 bases in the Philippines. Eligible participants from communities participating in the Transform Program of ICM will receive either the intervention, consisting of a 10-minute educational video about the efficacy and safety of measles and polio vaccines and vaccination reminders, or standard program sessions delivering health education through participatory adult learning sessions. Primary outcomes will include measles vaccination rates among the children of the participating families, and secondary outcomes will include polio vaccination rates, participant knowledge, and attitudes toward vaccines. In addition, the feasibility of the intervention will also be assessed.

Our intervention aims to address the last mile challenge of reaching WHO vaccination targets by improving knowledge and awareness of measles and polio vaccines among the communities living in extreme poverty in the Philippines and to complement the continuing efforts of the DOH to improve measles and polio vaccination coverage. By adopting a community-based approach and embedding our intervention within an existing community-based program run by our local partner, we leverage the established trust between community members and health educators. This approach is expected to enhance the acceptability and effectiveness of our intervention, as community members are more likely to engage with familiar and trusted sources of information, namely, pastors and the nongovernmental organization facilitating the program. In addition, using local health educators and staff who have long-standing working relationships with these communities will facilitate cultural competency and help ensure that interventions are tailored to meet the unique needs of the target population. Evidence on the impact of the intervention and the factors influencing its impact will be generated from the trial, and local stakeholders will be informed about the sustainability and scalability of this intervention to other regions of the Philippines. By systematically documenting the outcomes and lessons learned, we seek to contribute valuable insights that can guide the development and implementation of future community-based interventions aimed at improving vaccination coverage and addressing health disparities among marginalized populations.

### Strengths and Limitations

Our trial has the following strengths. First, the use of a cluster randomized controlled design enhances the validity of the study, providing a rigorous framework to evaluate the effectiveness of the intervention. This design will enable us to draw robust conclusions about the impact of the education toolkit on vaccination rates and knowledge. Second, using an implementation science approach will offer valuable insights into the facilitators and barriers encountered during the implementation of the intervention. By understanding the contextual factors influencing the success of the intervention, local stakeholders will be able to make informed decisions about its implementation and sustainability. Finally, this trial leverages our strong ties with our local partners in the Philippines, which strengthens the relevance and impact of our findings. By prioritizing equitable collaboration and engaging local partners from the development phase of the intervention, we aim to ensure that the benefits of the intervention extend to the most vulnerable populations. This approach is expected to foster vaccine confidence and support inclusive vaccination programs that address the needs of all community members.

However, while the vaccination status will be retrieved from the DOH, there is a possibility of missing data on the children of study participants. In such cases, relying on self-reported vaccination status may introduce recall bias, potentially affecting the accuracy of the data. Due to constraints on survey length, only 10 questions related to measles and polio vaccines will be included in the assessment of participants’ knowledge and attitudes. This limited scope may not fully capture the complexity of participants’ beliefs and understanding regarding vaccination. Despite this limitation, careful selection of survey questions based on key indicators will help ensure that essential information is obtained, balancing the depth of inquiry with practical considerations. Second, our intervention will be implemented alongside the soap opera intervention of the Transform Program. If the soap opera intervention is effective, participants in the intervention arm may make greater investments in children’s education, social capital, health, hope, and economic conditions, which would positively impact their children’s vaccination rates. Because the control arms will receive the traditional Transform Program, which includes the same content, any additional effect would be attributable to the soap opera delivery method, which is part of our intervention. Finally, participants in both arms may miss the health education and vaccine sessions due to other commitments, which could reduce their level of engagement with the intervention and its overall effectiveness.

## Appendix

Multimedia Appendix 1 Interview guides.

Multimedia Appendix 2 SPIRIT checklist.

Multimedia Appendix 3 Peer-review report from the Operating Grant: COVID-19 Vaccine Confidence Committee, Canadian Institutes of Health Research (CIHR).

## Abbreviations

DOH: Department of Health
ICM: International Care Ministries
IPV: inactivated polio vaccine
MCV: measles-containing vaccine
OPV: oral polio vaccine
SPIRIT: Standard Protocol Items: Recommendations for Interventional Trials
WHO: World Health Organization

## Data Availability

The datasets generated or analyzed during this study will be available from the corresponding author on reasonable request.

## References

[1] Moss WJ (2017). Measles. Lancet.

[2] (2012). Global measles and rubella strategic plan: 2012-2020. World Health Organization.

[3] (2023). About global polio eradication. Centers for Disease Control and Prevention.

[4] Infobox on measles. Philippine Pediatric Society.

[5] (2021). Routine immunization for children in the Philippines. United Nations Children’s Fund Philippines.

[6] Domai FM, Agrupis KA, Han SM, Sayo AR, Ramirez JS, Nepomuceno R, Suzuki S, Villanueva AM, Salva EP, Villarama JB, Ariyoshi K, Mulholland K, Palla L, Takahashi K, Smith C, Miranda E (2021). Measles outbreak in the Philippines: epidemiological and clinical characteristics of hospitalized children, 2016-2019. Lancet Reg Health West Pac.

[7] Ylade MC (2018). Epidemiology of measles in the Philippines. Acta Med Philipp.

[8] Measles vaccination coverage. World Health Organization.

[9] The Associated Press (2021). WHO, UNICEF declare end of polio outbreak in the Philippines. CTV News.

[10] (2022). Philippines is top 5 country in the world with zero dose children. United Nations Children’s Fund Philippines.

[11] Ozawa S, Yemeke TT, Evans DR, Pallas SE, Wallace AS, Lee BY (2019). Defining hard-to-reach populations for vaccination. Vaccine.

[12] Gupta D, Hassan B, Agarwal A, Bhasin A (2019). Immunization campaigns: mitigating barriers - designing communication. Interações Sociedade E As Novas Modernidades.

[13] Bhattacharya S, Saleem SM, Shikha D, Gokdemir O, Mehta K (2021). Role of vaccine science diplomacy in low-middle-income countries for eradicating the vaccine-preventable diseases: targeting the “last mile”. J Family Med Prim Care.

[14] Ali HA, Hartner AM, Echeverria-Londono S, Roth J, Li X, Abbas K, Portnoy A, Vynnycky E, Woodruff K, Ferguson NM, Toor J, Gaythorpe KA (2022). Vaccine equity in low and middle income countries: a systematic review and meta-analysis. Int J Equity Health.

[15] Gao Y, Kc A, Chen C, Huang Y, Wang Y, Zou S, Zhou H (2020). Inequality in measles vaccination coverage in the "big six" countries of the WHO South-East Asia region. Hum Vaccin Immunother.

[16] Helleringer S, Abdelwahab J, Vandenent M (2014). Polio supplementary immunization activities and equity in access to vaccination: evidence from the demographic and health surveys. J Infect Dis.

[17] Hajizadeh M (2018). Socioeconomic inequalities in child vaccination in low/middle-income countries: what accounts for the differences?. J Epidemiol Community Health.

[18] Larson HJ, Hartigan-Go K, de Figueiredo A (2019). Vaccine confidence plummets in the Philippines following dengue vaccine scare: why it matters to pandemic preparedness. Hum Vaccin Immunother.

[19] Ota MO, Badur S, Romano-Mazzotti L, Friedland LR (2021). Impact of COVID-19 pandemic on routine immunization. Ann Med.

[20] Sharma M, Singh SK, Sharma L, Dwiwedi MK, Agarwal D, Gupta GK, Dhiman R (2021). Magnitude and causes of routine immunization disruptions during COVID-19 pandemic in developing countries. J Family Med Prim Care.

[21] Haldane V, Dodd W, Kipp A, Ferrolino H, Wilson K, Servano D, Lau LL, Wei X (2022). Extending health systems resilience into communities: a qualitative study with community-based actors providing health services during the COVID-19 pandemic in the Philippines. BMC Health Serv Res.

[22] Lau LL, Hung N, Go DJ, Choi M, Dodd W, Wei X (2022). Dramatic increases in knowledge, attitudes and practices of COVID-19 observed among low-income households in the Philippines: a repeated cross-sectional study in 2020. J Glob Health.

[23] Luu K, Brubacher LJ, Lau LL, Liu JA, Dodd W (2022). Exploring the role of social networks in facilitating health service access among low-income women in the Philippines: a qualitative study. Health Serv Insights.

[24] Can a soap opera help alleviate poverty?. International Care Ministries.

[25] 2014 global report on poverty measurement with the progress out of poverty index. Poverty Probability Index.

[26] (2024). Media consumption in the Philippines: top platforms, trends in 2025. NGP Integrated Marketing Communications.

[27] (2023). Immunization dashboard Philippines. World Health Organization.

[28] Braun V, Clarke V, Hayfield N, Davey L, Jenkinson E, Bager-Charleson S, McBeath A (2023). Doing reflexive thematic analysis. Supporting Research in Counselling and Psychotherapy: Qualitative, Quantitative, and Mixed Methods Research.

[29] Braun V, Clarke V (2023). Toward good practice in thematic analysis: avoiding common problems and be(com)ing a researcher. Int J Transgend Health.

