# Supplementary material for: Effectiveness of an Education Toolkit Delivered by Soap Operas Among Communities Living in Extreme Poverty in Improving Vaccination Confidence in the Philippines: Protocol for a Cluster Randomized Controlled Trial
Source: JMIR Res Protoc. 2025 Oct 30;14:e77022. doi: 10.2196/77022 (PMC12616188; doi:10.2196/77022)
Supplement: Multimedia Appendix 1 [file resprot_v14i1e77022_app1.pdf]

Project Title: A cluster randomized controlled trial to evaluate the effectiveness of an educational toolkit among poor communities in improving vaccination confidence in the Philippines.

Interview Guide: End User Interview (**Health Trainers**)

Introductory questions

1. Please tell us your name, age, and what you do in the Transform Program?
2. What are your daily responsibilities as a health trainer?
3. How long you have been working in this role?

Vaccination barriers and facilitators

4. How much do people in the area know about vaccinations, especially MMR and polio vaccines?
5. Where do community members look for information about vaccinations?
    a. Are there certain places that people trust for vaccination information?
    b. How important do people in the community think vaccinations are?
    c. Are there cultural or social factors affecting community feelings towards vaccinations?
    d. Have you heard any wrong beliefs or myths about vaccinations in your community?
6. Approximately how many children (if any) did you notice were vaccinated with Polio and MMR vaccines in the communities you serve?
    a. Any campaigns from the DOH that promote the vaccination in communities?
7. What problems do community members face in getting their children vaccinated for polio and MMR? (For example, not knowing where to get vaccinations, cost, or distance from clinics)
8. How often do individuals in your community have their vaccination status recorded through vaccination cards?

Intervention

9. Can you share any challenges you face in teaching about vaccinations, and how do you handle them?
    a. How do you address situations where people have wrong beliefs about MMR (Measles, Mumps, Rubella) and polio vaccines or are resistant during your training sessions?
    b. Are there specific cultural values you consider when talking about vaccination education in the community?
    c. How do you ensure the effective communication of vaccination information, particularly to faith communities you work with?
10. Does the vaccine toolkit video help with community worries about MMR and polio shots?
    a. What information or messages in the video did you think to be most useful?
    b. Were the MMR and polio reminders helpful?
    c. Are there things missing in the video that are important for your community?
11. How did the community react to the lessons about MMR and polio vaccines shared in the video?
    a. How can the MMR/polio video be changed for the general public?
    b. Do you have any suggestions for improving the information?
    c. Do you have any suggestions for ways of sharing the information?
    d. Are there particular subjects or areas that you believe we should include or provide more information on during the educational sessions, such as health, children, or economic topics?
12. How do you feel about the workload you have as a health educator in the Transform Program?
13. Can you explain the training you got from ICM for the Transform Program and share how the coordinators have kept you updated throughout the implementation?
14. How do you think the toolkit lessons can be more integrated into the Transform Program?
    a. Are there any cultural or logistical considerations we should be aware of?
15. Do you think this toolkit could be used in the other regions of the Philippines?
    a. If yes – how might this be completed? Who would need to be involved?

b. If no – why not? What problems do you see in trying to implement?

Closing Questions

16. Do you have any final thoughts that you'd like to share?

Project Title: A cluster randomized controlled trial to evaluate the effectiveness of an educational toolkit among poor communities in improving vaccination confidence in the Philippines.

Interview Guide: End User Interview (**Community Members**)

Introductory questions

1. Please tell us your name, age, and what motivated you to participate in the Transform Program's efforts to educate about MMR and polio vaccinations?
2. What do you do for work?
3. What is your highest level of schooling?
4. How large is your family? How many children?
5. Have your children received polio and MMR vaccination?
    a. Was it before or after you attended the Transform Program?
6. How much did you know about MMR and polio vaccines before attending the Transform Program? Do you think they are important?
    a. Do your family and friends talk about these vaccines?
7. Where do you and your family and friends look for information about vaccinations?
    a. Are there certain places that you trust for vaccination information?
    b. How important do you think vaccinations are?
    c. Does anything influence your feelings towards vaccinations?
    d. Have you heard any wrong beliefs or myths about vaccinations?

For intervention arm only

8. After you attended the Transform program and watched the videos about polio and MMR vaccines, how did the educational video help you understand more about these vaccinations?
    a. Is the information from the video easy to understand and useful?
9. Did the video change your thoughts about vaccinations like MMR and polio?
    a. If yes, how?
    b. If no, why not?
10. Do you feel more confident or reassured about vaccinations for your children after participating in the Transform Program?
11. What do you think about the reminder messages about MMR and polio vaccines? Are they useful?
12. Were the education sessions effective in dealing with your concerns about MMR and polio vaccines?
    a. If you children are not yet vaccinated, do you plan to have your children vaccinated after the education session?
13. Do you have any remaining questions about polio and MMR vaccines after watching the video? Anything else that you would like to know about these vaccines or other vaccines?
14. Any suggestions to improve the video or how the vaccination information is delivered?

For control arm only

15. What did you learn about vaccines from the education session at week 12?
    a. Did you learn anything about MMR and polio vaccines?
    b. How was the information about vaccines are delivered at the health education session at week 12?
16. Was the education session effective in dealing with your concerns about MMR and polio vaccines?
    a. Any remaining question about Polio and MMR vaccines?
17. Did you have your children vaccinated after the education session? Or do you plan to have your children vaccinated after the education session?
18. How do you think the education session can be improved?

Vaccination barriers and facilitators

19. What considerations are most important to you when deciding whether to vaccinate? (e.g., vaccine safety, effectiveness, faith, cost, easy access, whether it is required by the government, etc)
20. Did you face any challenges or obstacles in having your children receive their MMR and polio vaccinations? (e.g., clinics too far, cost, don't know about these vaccines)
    a. If yes, please share your experience.
21. Are you aware of any national campaigns to promote polio and MMR vaccination? E.g., healthcare workers or barangay workers come to the village to vaccinate children for free.

<u>Closing Questions</u>

22. Do you have any additional thoughts or feedback about the program or vaccinations that you would like to share?

Project Title: A cluster randomized controlled trial to evaluate the effectiveness of an educational toolkit among poor communities in improving vaccination confidence in the Philippines.

Interview Guide: End User Interview (**Health coordinators**)

Introductory questions

1. Please tell us your name, age, and your specific role at ICM and in the Transform Program?
2. Could you describe your daily responsibilities as a health coordinator and how long you have been working in this role?

Vaccination barriers and facilitators

3. Approximately how many children (if any) did you notice were vaccinated with Polio and MMR vaccines in the communities participating Transform Program?
4. How much do people in these communities know about vaccinations, especially MMR (Measles, Mumps, Rubella) and polio vaccines?
5. Where do community members look for information about vaccinations?
    a. Are there certain places that people trust for vaccination information?
    b. How important do people in the community think vaccinations are?
    c. Are there cultural or social factors affecting community feelings towards vaccinations?
    d. Have you heard any wrong beliefs or myths about vaccinations in your community?
6. What challenges do community members face in getting vaccinations?
    a. How does the cost of vaccination influence the decision to vaccinate children in your area?
    b. How easy is it for people to access vaccination services in your community?
    c. How do community beliefs or cultural practices affect the acceptance of polio and MMR vaccinations?
    d. Are there a lot of misconceptions and misinformation circulating in the communities?
7. Are there any specific changes in healthcare policies or practices that you feel could increase vaccination coverage?
8. There have been several rounds of national immunization campaigns for polio and MMR vaccination since 2019. Do you think they have effectively improved the vaccination coverage in these communities?
    a. If yes, any gaps?
    b. If no, anything you think community-based programs can do to facilitate government's efforts?
9. Are there any other support or interventions from the government or NGOs to encourage families to vaccinate their children against polio and MMR? If yes, can you share some examples?
    a. E.g., do local health care providers or barangay health workers provide education or share information about MMR or polio vaccines in the communities regularly?
10. What role do you think community engagement plays in improving vaccination rates? Any other strategies that you think may be helpful to improve polio and MMR vaccination?
11. How much do community members get to decide about vaccinations?
12. How often do individuals in your community have their vaccination status recorded through vaccination cards?

Intervention

13. Does the video about MMR and polio vaccines help with community worries about MMR and polio shots?
    a. What information or messages in the video did you think to be most useful?
    b. Are there things missing in the video that are important for your community?
14. How do you think the tool kit (including the video and the lessons) can be more integrated into the Transform Program?

   a. Are there any cultural or logistical considerations we should be aware of?
   b. Any areas for improvement? Like the content or the ways of delivery?

15. Do you think this toolkit could be used in the other regions of the Philippines?
    a. If yes – how might this be completed? Who would need to be involved?
    b. If no – why not? What problems do you see in trying to implement the tool kit (people, money, equipment, testing centres, information)?
16. How do you ensure the effective communication of vaccination information, particularly to faith communities?
    a. Can you share any challenges you face in teaching about vaccinations, and how do you handle them?
    b. Additionally, could you describe your strategies for addressing misconceptions or resistance about vaccinations during your training sessions?

Closing Questions

17. Do you have any final thoughts that you'd like to share?
