# Supplementary material for: Effectiveness of an Education Toolkit Delivered by Soap Operas Among Communities Living in Extreme Poverty in Improving Vaccination Confidence in the Philippines: Protocol for a Cluster Randomized Controlled Trial
Source: JMIR Res Protoc. 2025 Oct 30;14:e77022. doi: 10.2196/77022 (PMC12616188; doi:10.2196/77022)
Supplement: Multimedia Appendix 3 [file resprot_v14i1e77022_app3.pdf]

Review Type/Type d'évaluation: Committee Member 1/Membre de comité 1
Name of Applicant/Nom du chercheur: Wei, Xiaolin
Application No./Numéro de demande: 469081
Agency/Agence: CIHR/IRSC
Competition/Concours: 2021-08-10 Operating Grant: COVID-19 Vaccine Confidence/Subvention de fonctionnement : Confiance à l’égard des vaccins contre la COVID-19
Committee/Comité: Operating Grant : COVID-19 Vaccine Confidence/Subvention de fonctionnement : Confiance à l’égard des vaccins contre la COVID-19
Title/Titre: Developing a tool kit to build vaccine confidence and ensure equitable COVID-19 vaccination implementation strategies in low-and-middle income countries: a multi-stakeholder implementation science and co-development study

---

Assessment/Évaluation:

Research Approach

Overall strengths and weaknesses and explanation of rating: This proposal aims to explore vaccine delivery strategies to at-risk and underserved groups in the Philippines, with a goal of enhancing vaccine confidence and creating an implementation tool kit to guide key stakeholders in implementing equitable COVID-19 vaccination programs in the Philippines and other LMICs. It is novel among applications because of its focus on LMICs. Strengths of this proposal include that it engages an equity approach from the beginning and engages community members in all aspects of study and development of the toolkit. Weaknesses of the project include that the data collection uses only qualitative methods, particularly when a survey or some other brief quantitative approach would really strengthen the ability of the researchers to detect any effect during roll out. The proposed research is likely to be able to address both gaps in knowledge (Objectives 1 and 2) and vaccine confidence (particularly Objective 5), although the follow-on project that may result will do a better job of getting at population level impacts. The use of purely qualitative methods limits the potential of this project. It makes sense to do interviews with stakeholders in the development of the toolkit, although a sample of 30 might be too limited to develop a robust tool kit. However, I assessing the impact of it, doing another 30 purposive interviews among end users won’t really be able to hint at effectiveness. The proposed scope of work is highly feasible in the 2 years and is likely to inform action on COVID-19. The engagement strategy is well articulated and broad (include WHO, etc.) and is highly likely to result in uptake of the toolkit upon completion of the project. Their mobilization plan leverages ongoing research in the Philippines to give the project a jump start. They will work with HCWs in a provincial or municipal hospital, a city health unit (primary care providers), a barangay health station (community care), our partner NGO (community care), and a private hospital. This mix of settings is a strength. With regard to health equity integration, their work is guided by the health equity implementation framework so equity highly integrated, in addition, the operationalization of this framework is a strength. However, the specification of the “marginalized groups” who are the intended audience is underspecified. Their equity plan is well specified and elements of it are carried throughout the proposal.

Applicants

Overall Strengths and weaknesses and explanation of rating: The proposed team is a multi-national research team that involves international and local experts and practitioners with expertise in public health, vaccine hesitancy research, implementation science, social science, and applied experience in low- and middle-income countries (LMICs). The PI is a policy expert, which will help ensure that the results are translated into actionable terms at the end of the funding. The team is comprised of diverse members in terms of discipline, gender and culture. Many researchers in the team have extensive experiences in global health and established long-term working relationships with researchers and organizations in LMICs. The team has a

**Review Type/Type d'évaluation:**	Committee Member 1/Membre de comité 1
**Name of Applicant/Nom du chercheur:**	Wei, Xiaolin
**Application No./Numéro de demande:**	469081
**Agency/Agence:**	CIHR/IRSC
**Competition/Concours:**	2021-08-10 Operating Grant: COVID-19 Vaccine Confidence/Subvention de fonctionnement : Confiance à l’égard des vaccins contre la COVID-19
**Committee/Comité:**	Operating Grant : COVID-19 Vaccine Confidence/Subvention de fonctionnement : Confiance à l’égard des vaccins contre la COVID-19
**Title/Titre:**	Developing a tool kit to build vaccine confidence and ensure equitable COVID-19 vaccination implementation strategies in low-and-middle income countries: a multi-stakeholder implementation science and co-development study

Assessment/Évaluation:

strong history of demonstrated support demonstrated established partnerships with the International Care Ministries (ICM), a large NGO with extensive community networks for health education and poverty reduction reaching over 3 million people in the Philippines which will ensure resource mobilization and community engagement. The team is likely to be able to quickly mobilize because they have existing relationships with the organizations on the ground (and a strong letter of support) and existing connections to sites that will host the study.

Impact of the Research

Overall Strengths and weaknesses and explanation of rating: This research is likely to achieve actionable evidence to improve COVID-19 vaccine confidence and uptake. This research is likely to improve health equity by building capacity on the ground. Notably, their collective making process will ensure that the tool kits for equitable vaccine delivery are “made by LMICs for LMICs” and offer both a tool and a way to build capacity among local partners. The methodology of the proposed project is underpinned by principles of community engagement and collective making, which is a deeper form of co-design that allows knowledge to surface and be synthesized amongst a group. This approach will facilitate the strengthening of existing relationships and will allow the investigators to engage key stakeholders of COVID-19 vaccine implementation programs in the Philippines in each phase of the project.

Budget

Overall Strengths and weaknesses and explanation of rating: The budget Is adequate. A notable strength is that it includes funding for postdocs and graduate students, further demonstrating the potential for the reach of this research beyond the current funding opportunity. The budget for travel seems too small for the scope of the work in the Philippines.

Review Type/Type d'évaluation: Committee Member 2/Membre de comité 2
Name of Applicant/Nom du chercheur: Wei, Xiaolin
Application No./Numéro de demande: 469081
Agency/Agence: CIHR/IRSC
Competition/Concours: 2021-08-10 Operating Grant: COVID-19 Vaccine Confidence/Subvention de fonctionnement : Confiance à l'égard des vaccins contre la COVID-19
Committee/Comité: Operating Grant : COVID-19 Vaccine Confidence/Subvention de fonctionnement : Confiance à l'égard des vaccins contre la COVID-19
Title/Titre: Developing a tool kit to build vaccine confidence and ensure equitable COVID-19 vaccination implementation strategies in low-and-middle income countries: a multi-stakeholder implementation science and co-development study

---

Assessment/Évaluation:

Research Approach

Strengths:
The proposal focuses on an underserved large population with a view to identifying subpopulations requiring specific focus. It is also a notable strength that the applicants are thinking of taking the general learning points beyond the immediate study context to allow upscaling of the evidence.

The proposal shows good organisation around a guiding framework and mapping of phases onto the framework. Overall there is a good use of existing approaches and frameworks grounding the research in existing methodologies.

The proposal is relatively clear in the overall steps that will be taken in the research process.

Weaknesses:
The proposal mentions "barriers, enablers, and 'key ingredients'" a lot without providing detail as to what this refers to – Barriers to what? Enablers of what? What are 'key ingredients'?

Additionally, given that the proposal uses an "implementation science approach" it is surprising to see no theory being mentioned, as is customary in implementation science research. There are plenty of theories which provide guidance on the factors relevant to the implementation process, which are not explicitly mentioned in the proposal. Using these might have provided more concrete information on the barriers and enablers narrative.

It would have been nice to give an example of what type of tool the applicants might consider developing. The tool references throughout the proposal remain in the abstract only, making it difficult to picture concrete interventions.

The proposal remains vague in places on who specifically will be targeted and the number of participants for some of the specific research activities. For example, how many participants will participate in the 'collective making' approach and who are likely target populations?

Quality of Applicants
The applicants appear to be qualified to carry out the proposed work. There are no obvious gaps in the skillset of the team.

**Review Type/Type d'évaluation:**	Committee Member 2/Membre de comité 2
**Name of Applicant/Nom du chercheur:**	Wei, Xiaolin
**Application No./Numéro de demande:**	469081
**Agency/Agence:**	CIHR/IRSC
**Competition/Concours:**	2021-08-10 Operating Grant: COVID-19 Vaccine Confidence/Subvention de fonctionnement : Confiance à l'égard des vaccins contre la COVID-19
**Committee/Comité:**	Operating Grant : COVID-19 Vaccine Confidence/Subvention de fonctionnement : Confiance à l'égard des vaccins contre la COVID-19
**Title/Titre:**	Developing a tool kit to build vaccine confidence and ensure equitable COVID-19 vaccination implementation strategies in low-and-middle income countries: a multi-stakeholder implementation science and co-development study

Assessment/Évaluation:

Impact of the research

The research shows promise for impact in both the study context as well as beyond. The applicants have thought about how the generic evidence can be used in other comparable context which increases the likelihood of producing interventions that can be upscaled beyond the period of the current project.

Budget

The budget seems appropriate for the proposed work.

Review Type/Type d'évaluation: Committee Member 3/Membre de comité 3
Name of Applicant/Nom du chercheur: Wei, Xiaolin
Application No./Numéro de demande: 469081
Agency/Agence: CIHR/IRSC
Competition/Concours: 2021-08-10 Operating Grant: COVID-19 Vaccine Confidence/Subvention de fonctionnement : Confiance à l'égard des vaccins contre la COVID-19
Committee/Comité: Operating Grant : COVID-19 Vaccine Confidence/Subvention de fonctionnement : Confiance à l'égard des vaccins contre la COVID-19
Title/Titre: Developing a tool kit to build vaccine confidence and ensure equitable COVID-19 vaccination implementation strategies in low-and-middle income countries: a multi-stakeholder implementation science and co-development study

---

Assessment/Évaluation:

The focus of the proposal is on an implementation science study for strategies that mitigate vaccine hesitancy in LMICS. The team is proposing to develop these strategies by engaging a partner in The Philippines.

The team hopes that the information learned from this specific context could be applicable to other contexts. While, their main objective is to develop a "country-specific" tool kit that reflects the specific contextual factors, they would also develop a generic tool kit for adaptation elsewhere.

The main research team will be based in Canada relying heavily on the groundwork from the main local partner. The proposal indicates that they will work with local hospitals, city health unit, barangay health station and a private hospital; however, letters of support or collaboration would be helpful to better understand how they are positioned to contribute to the project.

The team plans to conduct a grey literature review and qualitative interviews with a wide range of stakeholders, with an emphasis on representatives from organizations. The extent to which and how community members are fully included in this phase of the project or for that matter throughout the life of the project is not described. Further, who and how interviews will occur and the data analysis plan lack description. This is similar to the evaluation of the pilot of the toolkit.

The toolkit will be developed and piloted with trainees with an evaluation that focuses on the immediate learning outcomes and perception of relevance.

There is limited inclusion of diversity across socio-cultural groups that are to benefit from the toolkit. The proposal includes a description of how sex and gender would be considered; however, there is a clear conflation between gender as to mean "women."
